# Entanglement generation between different center-of-mass motions in double levitated micromagnet system

**DOI:** 10.1038/s41598-025-28242-9

**Published:** 2025-12-29

**Authors:** Wenjie Liu, Shi Rao

**Affiliations:** 1 School of Science, Hubei University of Technology, Wuhan, 430068 China; 2 Key Laboratory of Quantum Information, University of Science and Technology of China, Hefei, 230026 China

**Keywords:** Optics and photonics, Physics

## Abstract

We theoretically propose a scheme to realize quantum entanglement between two center-of-mass motion (CM) as the external degrees of freedom of two separated levitated yttrium iron garnet spheres. The microwave cavity field is introduced to bridge the interaction between the two separated micromagnet spheres. The optimal condition for getting entanglement between the two CM modes with and without effective cavity-magnon interaction cases are investigated. It is found that the entanglement between two CM modes could be obtained by choosing the appropriate magnon detunings for each case. The effect of the coupling strength and the thermal occupation on the entanglement are also discussed. To realize the entanglement between to CM modes, it is necessary to balance the related parametric interaction and beam splitter interaction. The results show that the CM-CM entanglement is robust against the thermal noise. This scheme would be valuable for quantum network generation and quantum information processing.

## Introduction

Entanglement, a nonlocal correlation first proposed by Einstein as a “spooky action at a distance”^1,2^, has gained great attention in quantum physics and quantum optics^3,4^. Taking advantage of its nonclassical features^5^, entanglement has been proposed as a vital resource for quantum communication^6–8^, quantum computing^9–11^, quantum memory^12–14^, quantum metrology^15^, and quantum networking^16,17^. Until now, many investigations have been devoted to manipulate and investigate entanglement in microscopic systems and mesoscopic systems, such as the photons^18–20^, the ions^21,22^ and the superconduction system^23,24^. For further practical application, a key issue is to generate and manipulate entanglement in macroscopic massive systems. Entanglement in macroscopic systems is not only a versatile tool in quantum technology but also a subtle probe to explore the boundary between classical and quantum physics. Diverse methods have been proposed to generate entanglement in massive macroscopic systems, such as the engineered reservoir^8,25,26^, the injection of squeezed vacuum^27,28^ and the modulation of the pump^29,30^.

On the other hand, the cavity magnomechanical (CMM) system^31–33^ emerges as a fascinating platform to investigate the light-mater interaction on the macroscopic scale. Conventional CMM system are composed of ferromagnetic crystal with collective spin excitations and microwave cavity field. The magnon mode (collective spin excitation) in the ferromagnetic crystal such as yttrium iron garnet (YIG) can couple with the microwave photons through magnetic dipole interaction and the deformation phonon modes through mangetostrictive interaction^34,35^. Due to various advantages such as high spin density^32,36^, low dissipation rate^37,38^, wide frequency tunability^39^, desirable coherence time^40^, CMM system has been studied theoretically and experimentally for the generation of entanglement^33,41,42^. Meanwhile, by coupling additional atoms^43,44^, superconducting qubit^45^ and NV center^46^, hybrid CMM systems show diverse types of nonlinearity and become a versatile platform for quantum coherence establishment^43,44^ and quantum sensing^47^. In particular, by employing different types of nonlinearity, numerous schemes have been proposed to obtain entanglement between two massive mechanical modes^48–51^.

Notably, in a recent article, Kani et al. proposed an intensive CMM system where a magnetic particle can be trapped and levitated in a microwave cavity^52–54^. This levitated CMM system shows an additional external degree of freedom, the CM motion of a YIG sphere. Different from the conventional CMM system, the coupling strength in the levitated CMM system is independent of the mass and size of the YIG sphere and convenient for the generation and utilization of entanglement at macroscopic scale. Bayati et al.^55^ found that the magnon mode as an internal degree of freedom can get entangled with the CM motion as an external degree of freedom by driving the magnon mode with parametric amplification. Yang et al.^56^ discovered that bipartite and tripartite entanglement in the intensive CMM system could be established by modulating the cavity and magnon detunings appropriately. Then, the characteristics of bipartite and tripartite entanglement were investigated, respectively, in the hybrid levitated CMM system with Coulomb interaction and the double-cavity levitated CMM system. Zhang et al.^57^ studied the generation of tripartite entanglement in a levitated CMM system with a nonlinear optical medium. Previously, these studies mainly focused on the establishment of entanglement between the internal degree of freedom and the external degree of freedom. It seems to us that the entanglement between two CM motions as the external degree of freedom is still lacking. Motivated by these results, we wondered whether it is possible to generate massive entanglement between two macroscopic CM motions.

In this paper, we want to generate the entanglement between two CM motions via an intermediated cavity field. There are three merits related to this research. First, the dissipation rate of the CM mode is relatively smaller than that of the conventional acoustic phonon mode. Therefore, the quantum entanglement prepared in this levitated CMM system can exist for a longer period of time. Second, the coupling coefficient between the cavity field and the magnon mode in levitated CMM system is independent of the size and mass of the YIG sphere. For conventional CMM systems, the interaction between the cavity and the mechanical mode originates from the deformation vibration and decreases as the size increases. In levitated CMM systems, the coupling between the cavity field and the mechanical modes does not change with size and can be adjusted through parameters such as the pump field and the position of the YIG sphere. This is very beneficial for the preparation and control of entanglement of macroscopic particles of large size. Third, the CM motion is favorable for optical detection. The study based on entanglement between different CM modes may offer a potential for the visual presentation of macroscopic quantum phenomena. After systematic analysis, we found that the two CM motions can be entangled when the detunings of the cavity field and the magnon mode are controlled appropriately. We analyze the effect of coupling strength and thermal occupation on entanglement. The entanglement we generated is robust to thermal occupation. In addition, the mechanism behind the generation of entanglement is studied.

The remainder of the paper is structured as follows. In the second section, we present the system schematic and the related Hamiltonian. In the third section, we analyze the effect of the system parameters on the entanglement between two CM modes. The last section presents the conclusion.

## The model and system Hamiltonian

**Fig. 1:**
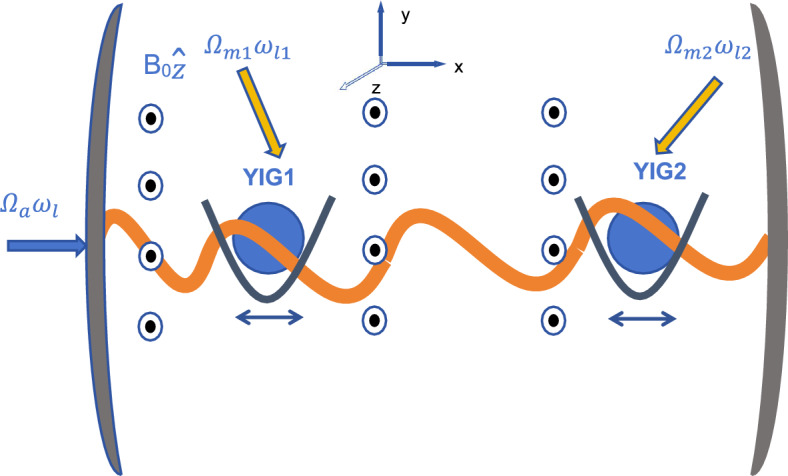
The schematic of the cavity magnomechanical system with double levitated YIG spheres. YIG levitated spheres are trapped in in a harmonic potential along the x direction of the microwave-wave axis. The YIG spheres and the cavity are driven by external fields. A uniform bias magnetic field along the *z* direction of the cavity axis is exerted to realize the magnon-photon interaction.

In this section, we consider a hybrid CMM system with double levitated YIG spheres. As shown in Fig. 1, the CM motions of the two YIG spheres are trapped in a harmonic oscillator potential. The magnon modes in the YIG spheres are excited by homogeneous magnetic fields and then coupled to the cavity field through the magnetic dipole interaction^58,59^. The Hamiltonian of the system is given below^52^

1 $$\begin{aligned} H/\hbar&=\omega _{a}a^{\dagger }a+\sum _{j=1,2}[\omega _{mj}m_{j}^{\dagger }m_{j}+\frac{\omega _{cj}}{2}(x_{j}^{2}+p_{j}^{2})+g_{amj}(am_{j}^{\dagger }+a^{\dagger }m_{j})\cos (kx_{j})]\nonumber \\&\quad +\Omega _{a}(a e^{i\omega _{d}t}+a^{\dagger }e^{-i\omega _{d}t})+\sum _{j=1,2}\Omega _{mj}(m_{j}e^{i\omega _{lj}t}+m_{j}^{\dagger }e^{-i\omega _{lj}t}). \end{aligned}$$

The first three terms describe the free Hamiltonian of the cavity mode, the magnon modes, and the CM motions of levitated micromagnetic particles. Here *a*($a^{\dag }$) and $m_{j}$($m^{\dag }_{j}$) denote the annihilation (creation) operators of the cavity field (with frequency $\omega _{a}$) and the *j*-th magnon mode (with frequency $\omega _{mj}$), respectively. $x_{j}$ and $p_{j}$ are the position and momentum operators of the *j*-th magnetic particle CM motion with oscillating frequency $\omega _{cj}$. The fourth term $g_{amj}(am_{j}^{\dagger }+a^{\dagger }m_{j})\cos (kx_{j})$ is related to the magnetic dipole interaction between the *j*-th magnon mode and the cavity field with coupling coefficients $g_{amj}=\frac{\gamma }{2}\sqrt{\frac{\hbar \omega _{a}\mu _{0}}{V_{a}}}\sqrt{2\rho _{s}V_{j}s}$ ^60^. Here, $\gamma$ denotes the gyromagnetic ratio. $\mu _{0}$ represents the vacuum permeability. $V_{a}$ and $V_{j}$ correspond to the mode volumes of the cavity and the *j*-th YIG sphere, respectively. $\rho _{j}$ is the related spin density of the *j*-th YIG sphere. $s=\frac{5}{2}$ is the spin number of the ground state of the YIG spheres. The fifth and sixth terms of the Hamiltonian describe the driving terms of the cavity field and the *j*-th magnon mode with driving strength $\Omega _{a}=\sqrt{\frac{2P_{a}\kappa _{a}}{\hbar \omega _{d}}}(\Omega _{mj}=\sqrt{\frac{2P_{mj}\kappa _{mj}}{\hbar \omega _{lj}}})$ and frequency $\omega _{d}$ and $\omega _{lj}$, respectively. Given the fact that the thermal variance of the CM position of the *j*-th YIG sphere $\Delta x_{j}$ is much smaller than the microwave wavelength, we can expand $\cos (kx_{j})$ up to the first order in $x_{j}$ around the minimum position of the trap $x_{j0}$, that is, $\cos {kx_{j}}=\cos {kx_{j0}}-k\sin {kx_{j0}}(x_{j}-x_{j0})$. By defining the relative position and momentum of the *j*-th CM mode as $x_{j}-x_{j0}=\frac{1}{2}(c_{j}+c_{j}^{\dagger })$ and $p_{j}=\frac{1}{2i}(c_{j}-c_{j}^{\dagger })$, we can rewrite the Hamiltonian (1) with the annihilation operator $c_{j}$ (creation operator $c_{j}^{\dagger }$) of the *j*-th CM mode as follows^52,54,55^

2 $$\begin{aligned} H/\hbar&=\omega _{a}a^{\dagger }a+\sum _{j=1,2}[\omega _{mj}m_{j}^{\dagger }m_{j}+\frac{\omega _{cj}}{2}c_{j}^{\dagger }c_{j}+g_{amj}(am_{j}^{\dagger }+a^{\dagger }m_{j})\cos (kx_{j0})]\nonumber \\&\quad -\sum _{j=1,2}g_{amcj}(am_{j}^{\dagger }+a^{\dagger }m_{j})\sin (kx_{j0})(c_{j}+c_{j}^{\dagger })+\Omega _{a}(a e^{i\omega _{d}t}+a^{\dagger }e^{-i\omega _{d}t})\nonumber \\&\quad +\sum _{j=1,2}\Omega _{mj}(m_{j}e^{i\omega _{lj}t}+m_{j}^{\dagger }e^{-i\omega _{lj}t}), \end{aligned}$$

with $g_{amcj}=g_{amj}k/2$. *k* denotes the microwave wave number^52^. To simplify the calculation and analysis in the following part, we assume that $\omega _{d}=\omega _{lj}$. Applying the unitary transformation $U=\exp {(-i\tilde{H}_{0}t/\hbar )}$ with $\tilde{H}_{0}=\hbar \omega _{d}(a^{\dagger }a+m_{1}^{\dagger }m_{1}+m_{2}^{\dagger }m_{2})$, the effective Hamiltonian can be derived as

3 $$\begin{aligned} H^{\prime }/\hbar&=U^{\dagger }HU/\hbar +i\frac{dU^{\dagger }}{dt}U\nonumber \\&=\Delta _{a}a^{\dagger }a+\sum _{j=1,2}[\Delta _{mj}m_{j}^{\dagger }m_{j}+\frac{\omega _{cj}}{2}c_{j}^{\dagger }c_{j}+g_{amj}(am_{j}^{\dagger }+a^{\dagger }m_{j})\cos (kx_{j0})]\nonumber \\&\quad +\sum _{j=1,2}g_{amcj}(am_{j}^{\dagger }+a^{\dagger }m_{j})\sin (kx_{j0})(c_{j}+c_{j}^{\dagger })+\Omega _{a}(a+a^{\dagger })\nonumber \\&\quad +\sum _{j=1,2}\Omega _{m_{j}}(m_{j}+m_{j}^{\dagger }), \end{aligned}$$

with $\Delta _{a}=\omega _{a}-\omega _{d}$ ($\Delta _{mj}=\omega _{mj}-\omega _{d}$) representing the detuning of the cavity (the *j*-th magnon excitations) with respect to the driving field.

Assuming that the interaction between the system and the environment does not lead to memory effects, we can obtain the system quantum Langevin equations under Markov approximation as follows,

4 $$\begin{aligned} \dot{a}&=-(\kappa _{a}+i\Delta _{a})a+\sum _{j=1,2}[-ir_{1j}m_{j}+ir_{2j}m_{j}(c_{j}+c_{j}^{\dagger })]-i\Omega _{a}+\sqrt{2k_{a}}a_{in},\nonumber \\ \dot{m_{j}}&=-(\kappa _{mj}+i\Delta _{mj})m_{j}-ir_{1j}a+ir_{2j}a(c_{j}+c_{j}^{\dagger })-i\Omega _{mj}+\sqrt{2k_{mj}}m_{j,in},\nonumber \\ \dot{c_{j}}&=-(\gamma _{cj}+i\omega _{cj})c_{j}+ir_{2j}(a^{\dagger }m_{j}+am_{j}^{\dagger })+\sqrt{2\gamma _{cj}}c_{j,in}, \end{aligned}$$

where $\kappa _{a}$, $\kappa _{mj}$, and $\gamma _{cj}$ are the decay rates of the microwave mode, *j*-th magnon mode, and *j*-th CM mode, respectively. $r_{1j}=g_{amj}cos(kx_{j0})$ is the effective coupling coefficient between the cavity field and the magnon modes. $r_{2j}=g_{amcj}sin(kx_{j0})$ i.e., the related coupling strength of the tripartite cavity-magnon-CM interaction. $a_{in}$, $m_{j,in}$, and $c_{j,in}$ are the corresponding input quantum noise operators. These noise operators have zero mean values and satisfy the following correlation functions,

5 $$\begin{aligned}<a_{in}(t)a_{in}^{\dagger }(t^{\prime })>&=(\bar{n}_{a}+1)\delta (t-t^{\prime }),\nonumber \\<a_{in}^{\dagger }(t)a_{in}(t^{\prime })>&=\bar{n}_{a}\delta (t-t^{\prime }),\nonumber \\<m_{j,in}(t)m_{j,in}^{\dagger }(t^{\prime })>&=(\bar{n}_{mj}+1)\delta (t-t^{\prime }),\nonumber \\<m_{j,in}^{\dagger }(t)m_{j,in}(t^{\prime })>&=\bar{n}_{mj}\delta (t-t^{\prime }),\nonumber \\<c_{j,in}(t)c_{j,in}^{\dagger }(t^{\prime })>&=(\bar{n}_{cj}+1)\delta (t-t^{\prime }),\nonumber \\ <c_{j,in}^{\dagger }(t)c_{j,in}(t^{\prime })>&=\bar{n}_{cj}\delta (t-t^{\prime }), \end{aligned}$$

with $\bar{n}_{\vartheta } = 1/[exp(\hbar \omega _{\vartheta }/k_{B}T)-1] (\vartheta =a,mj,cj)$ as the related thermal number. $k_{B}$ and *T* are the Boltzmann constant and the environmental temperature.

Since we are interested in the quantum correlation between the fluctuation parts of different subsystems, we adopt the standard linearization operation in dynamic analysis to the quantum Langevin equations (4). Each operator can be decomposed as the sum of a classical steady-state part and a fluctuation part, that is, $\vartheta =\vartheta _{0}+\delta \vartheta (\vartheta =a,m_{j},c_{j})$. The quantum Langevin equations of the steady state mean values part are given as below

6 $$\begin{aligned} \dot{a_{0}}&=-(k_{a}+i\Delta _{a})a_{0}-\sum _{j=1,2}i(r_{1j}-2r_{2j}Re[c_{j0}])m_{j0}-i\Omega _{a},\nonumber \\ \dot{m_{j0}}&=-(k_{m_{j}}+i\Delta _{m_{j}})m_{j0}-i(r_{1j}-2r_{2j}Re[c_{j0}])a_{0}-i\Omega _{m_{j}},\nonumber \\ \dot{c_{j0}}&=-(\gamma _{c_{j}}+i\omega _{c_{j}})c_{j0}+ir_{2j}(a_{0}^{*}m_{j0}+a_{0}m_{j0}^{*}),(j=1,2). \end{aligned}$$

From the above equations, we can get the steady state values of the operators in the form as

7 $$\begin{aligned} a_{0}&=\frac{i\Omega _{a}t_{1}t_{2}+i\Omega _{m1}t_{1}G_{1}+i\Omega _{m2}t_{2}G_{2}}{-t_{0}t_{1}t_{2}-t_{1}G_{2}^{2}-t_{2}G_{1}^{2}},\nonumber \\ m_{j0}&=\frac{i\Omega _{a}t_{1}t_{2}G_{j}+i\Omega _{m1}t_{1}G_{1}G_{j}+i\Omega _{m2}t_{2}G_{2}G_{j}+i\Omega _{mj}(-t_{0}t_{1}t_{2}-G_{1}^{2}t_{2}-G_{2}^{2}t_{1})}{-t_{0}t_{1}t_{2}t_{j}-t_{1}t_{j}G_{2}^{2}-t_{2}t_{j}G_{1}^{2}},\nonumber \\ c_{j0}&=\frac{ir_{2j}(a_{0}^{*}m_{j0}+a_{0}m_{m0}^{*})}{\gamma _{cj}+i\omega _{cj}},(j=1,2), \end{aligned}$$

where $t_{0}=k_{a}+i\Delta _{a}$, $t_{1}=k_{m1}+i\Delta _{m1}$, $t_{2}=k_{m2}+i\Delta _{m2}$, $G_{1}=ir_{11}-ir_{21}(c_{10}+c^{*}_{10})$, $G_{2}=ir_{12}-ir_{22}(c_{20}+c^{*}_{20})$. By subtracting the steady-state parts from the operators, we can obtain the quantum Langevin equations of the fluctuation parts as

8 $$\begin{aligned} \dot{\delta a}&=-t_{0}\delta a+\sum _{j=1,2}[-iG_{j}\delta m_{j}+iG_{mj}(\delta c_{j}+\delta c_{j}^{\dagger })]+\sqrt{2k_{a}}a_{in},\nonumber \\ \dot{\delta m_{j}}&=-t_{j}\delta m_{j}-iG_{j}\delta a+iG_{aj}(\delta c_{j}+\delta c_{j}^{\dagger })+\sqrt{2k_{mj}}m_{j,in},\nonumber \\ \dot{\delta c_{j}}&=-(\gamma _{cj}+i\omega _{cj})\delta c_{j}+iG_{mj}(\delta a^{\dagger }+\delta a)+iG_{aj}(\delta m_{j}^{\dagger }+\delta m_{j})+\sqrt{2\gamma _{cj}}c_{j,in}. \end{aligned}$$

Here $G_{aj}=r_{2j}a_{0}$,$G_{mj}=r_{2j}m_{j0}$. The values of $G_{aj}$ and $G_{mj}$ can be adjusted by placing the trap at different nodes.

For the benefit of further analysis, we introduce the quadrature operators of microwave, magnon, and CM modes as $X_{\vartheta }=(\vartheta +\vartheta ^{\dagger })/\sqrt{2}$ and $Y_\vartheta =(\vartheta -\vartheta ^{\dagger })/\sqrt{2}i$, $\vartheta =a,m_{1},m_{2},c_{1},c_{2}$. The corresponding noise operators are defined as $X_{\vartheta }^{in}=(\vartheta _{in}+\vartheta _{in}^{\dagger })/\sqrt{2}$ and $Y_{\vartheta }^{in}=(\vartheta _{in}-\vartheta _{in}^{\dagger })/\sqrt{2}i$. From Eq. (8) we can obtain the Langevin equations of the quadrature operators $X_{\vartheta }$ and $Y_{\vartheta }$ as below

9 $$\begin{aligned} \dot{\delta X_{a}}&=-\kappa _{a}\delta X_{a}+\Delta _{a}\delta Y_{a}+G_{1}\delta Y_{m1}+G_{2}\delta Y_{m2}+\sqrt{2\kappa _{a}}X_{a}^{in},\nonumber \\ \dot{\delta Y_{a}}&=-\kappa _{a}\delta Y_{a}-\Delta _{a}\delta X_{a}-G_{1}\delta X_{m1}-G_{2}\delta X_{m2}+2G_{m1}\delta X_{c1}+2G_{m2}\delta X_{c2}+\sqrt{2\kappa _{a}}Y_{a}^{in},\nonumber \\ \dot{\delta X_{m1}}&=-\kappa _{m1}\delta X_{m1}+\Delta _{m1}\delta Y_{m1}+G_{1}\delta Y_{a}+\sqrt{2\kappa _{m1}}X_{m1}^{in},\nonumber \\ \dot{\delta Y_{m1}}&=-\kappa _{m1}\delta Y_{m1}-\Delta _{m1}\delta X_{m1}-G_{1}\delta X_{a}+2G_{a1}\delta X_{c1}+\sqrt{2\kappa _{m1}}Y_{m1}^{in},\nonumber \\ \dot{\delta X_{m2}}&=-\kappa _{m2}\delta X_{m2}+\Delta _{m2}\delta Y_{m2}+G_{2}\delta Y_{a}+\sqrt{2\kappa _{m2}}X_{m2}^{in},\nonumber \\ \dot{\delta Y_{m2}}&=-\kappa _{m2}\delta Y_{m2}-\Delta _{m2}\delta X_{m2}-G_{2}\delta X_{a}+2G_{a21}\delta X_{c2}+\sqrt{2\kappa _{m2}}Y_{m2}^{in},\nonumber \\ \dot{\delta X_{c1}}&=-\gamma _{c1}\delta X_{c1}+\omega _{c1}\delta Y_{c1}+\sqrt{2\gamma _{c1}}X_{c1}^{in},\nonumber \\ \dot{\delta Y_{c1}}&=-\gamma _{c1}\delta Y_{c1}-\omega _{c1}\delta X_{c1}+2G_{m1}\delta X_{a}+2G_{a1}\delta X_{m1}+\sqrt{2\gamma _{c1}}Y_{c1}^{in},\nonumber \\ \dot{\delta X_{c2}}&=-\gamma _{c2}\delta X_{c2}+\omega _{c2}\delta Y_{c2}+\sqrt{2\gamma _{c2}}X_{c2}^{in},\nonumber \\ \dot{\delta Y_{c2}}&=-\gamma _{c2}\delta Y_{c2}-\omega _{c2}\delta X_{c2}+2G_{m2}\delta X_{a}+2G_{a2}\delta X_{m2}+\sqrt{2\gamma _{c2}}Y_{c2}^{in}. \end{aligned}$$

By arranging the quadrature operators in the form of column vectors such as $u(t)=[\delta X_{a},\delta Y_{a},\delta X_{m1},\delta Y_{m1},\delta X_{m2},\delta Y_{m2},\delta X_{c1}$, $\delta Y_{c1},\delta X_{c2},\delta Y_{c2}]^{T}$, we can rewrite Eq.(9) for the quadrature operators in matrix form as

10 $$\begin{aligned} \dot{u(t)}=M(t)u(t)+n(t), \end{aligned}$$

with time-dependent drift matrix,

11 $$\begin{aligned} A=\begin{pmatrix} -\kappa _{a}& \Delta _{a}& 0& G_{1}& 0& G_{2}& 0& 0& 0& 0 \\ -\Delta _{a}& -\kappa _{a}& -G_{1}& 0& -G_{2}& 0& 2G_{m1}& 0& 2G_{m2}& 0 \\ 0& G_{1}& -\kappa _{m1}& \Delta _{m}& 0& 0& 0& 0& 0& 0 \\ -G_{1}& 0& -\Delta _{m1}& -\kappa _{m1}& 0& 0& 2G_{a1}& 0& 0& 0 \\ 0& G_{2}& 0& 0& -\kappa _{m2}& \Delta _{m}& 0& 0& 0& 0 \\ -G_{2}& 0& 0& 0& -\Delta _{m2}& -\kappa _{m2}& 0& 0& 2G_{a2}& 0 \\ 0& 0& 0& 0& 0& 0& -\gamma _{c1}& \omega _{c1}& 0& 0 \\ 2G_{m1}& 0& 2G_{a1}& 0& 0& 0& -\omega _{c1}& -\gamma _{c1}& 0& 0 \\ 0& 0& 0& 0& 0& 0& 0& 0& -\gamma _{c2}& \omega _{c2} \\ 2G_{m2}& 0& 0& 0& 2G_{a2}& 0& 0& 0& -\omega _{c2}& -\gamma _{c2} \end{pmatrix} \end{aligned}$$

and the noise column vector $n(t)=[\sqrt{2\kappa _{a}} X^{in}_{a},\sqrt{2\kappa _{a}} Y^{in}_{a},\sqrt{2\kappa _{m1}} X^{in}_{m1},\sqrt{2\kappa _{m1}} Y^{in}_{m1},\sqrt{2\kappa _{m2}} X^{in}_{m2},\sqrt{2\kappa _{m2}} Y^{in}_{m2},\sqrt{2\gamma _{c1}} X^{in}_{c1},\sqrt{2\gamma _{c1}} Y^{in}_{c1}$, $\sqrt{2\gamma _{c2}} X^{in}_{c2},\sqrt{2\gamma _{c2}} Y^{in}_{c2},]^{T}$.

Since we are interested in the quantum correlation of different subsystems, we would like to analyze the elements of the covariance matrix V defined as $V_{ij}=\langle u_{i}(t)u_{j}(t^{\prime })+u_{j}(t^{\prime })u_{i}(t)\rangle /2$. Following the fluctuation Langevin equations (8), we can get the Lyapunov equation^61,62^,

12 $$\begin{aligned} AV+VA^{T}=-D, \end{aligned}$$

where the diffusion matrix is $$D = diag\left[ {\kappa _{a} (2\bar{n}_{a} + 1),} \right.$$$$\kappa _{a} (2\bar{n}_{a} + 1),\kappa _{{m1}} (2\bar{n}_{{m1}} + 1),$$$$\kappa _{{m1}} (2\bar{n}_{{m1}} + 1),\kappa _{{m2}} (2\bar{n}_{{m2}} + 1),$$$$\kappa _{{m2}} (2\bar{n}_{{m2}} + 1),\kappa _{{c1}} (2\bar{n}_{{c1}} + 1),$$$$\kappa _{{c1}} (2\bar{n}_{{c1}} + 1),\left. {\kappa _{{c2}} (2\bar{n}_{{c2}} + 1),\kappa _{{c2}} (2\bar{n}_{{c2}} + 1)} \right]$$. To study the degree of bipartite entanglement, we adopt the quantum correlation indicator of logarithmic negativity $$E_{n}$$. For a bipartite system with a $$4\times 4$$ covariance matrix $$V_{\alpha \beta }=\left( \begin{array}{cc} V_{\alpha } & V_{\gamma } \\ V_{\gamma }^{T} & V_{B} \\ \end{array} \right)$$, logarithmic negativity $$E_{n}$$ is defined as

13 $$\begin{aligned} E_{n}=max[0,-ln(2\eta ^{-})], \end{aligned}$$

with $\eta ^{-}=2^{-1/2}[\Sigma -(\Sigma ^{2}-4\det V_{\alpha \beta })^{1/2}]^{1/2}$, $\Sigma =\det V_{\alpha }+\det V_{\beta }-2\det V_{\gamma }$. Here, $V_{\alpha }$,$V_{\beta }$, $V_{\gamma }$ represent the covariance matrix of the mode $\alpha$,$\beta$ and their correlations. The emergence of bipartite entanglement corresponds to $E_{n}>0$.

## Entanglement between two CM motions

In this section, we study the entanglements between two CM motions numerically. To simplify the discussion, we assume that the parameters of the two levitated YIG spheres are the same except their positions and pump fields. Hence $\omega _{c1}=\omega _{c2}=\omega _{c}$, $\omega _{m1}=\omega _{m2}=\omega _{m}$, $\gamma _{c1}=\gamma _{c2}=\gamma _{c}$, $\kappa _{m1}=\kappa _{m2}=\kappa _{m}$, $G_{a1}=G_{a2}=G_{a}$, $G_{m1}=G_{m2}=G_{m}$, $G_{1}=G_{2}=G_{0}$. The system parameters are chosen according to Ref.^56^: the normalized frequency of the CM oscillation frequency $\omega _{c}=1$, the damping rate of the magnon mode and the cavity mode is $\kappa _{a}=\kappa _{m}=0.1\omega _{c}$, the decay rate of the CM mode is $\gamma _{c}=10^{-5}\omega _{c}$, the coupling coefficients $G_{a}=G_{m}=0.08\omega _{c}$, the thermal occupation $\bar{n}_{c}=0.5$. The parameters are carefully chosen according to the Routh-Hurwitz criterion^63^ so that all the parameters of the system satisfy the stable conditions.

**Fig. 2:**
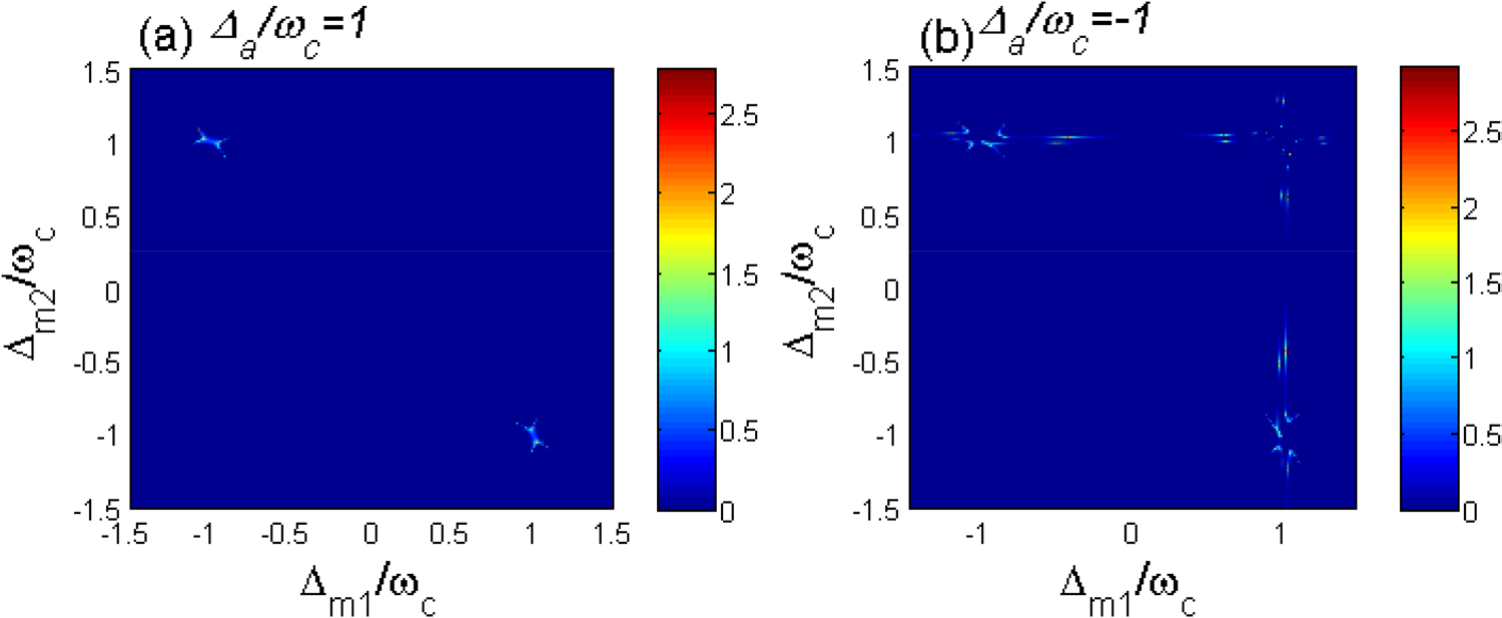
Phase diagram of the entanglement logarithmic negativity between the two CM motions as a function of $\Delta _{m1}/\omega _{c}$ and $\Delta _{m2}/\omega _{c}$ for (**a**) $\Delta _{a}/\omega _{c}=1$ and (**b**) $\Delta _{a}/\omega _{c}=-1$, respectively. The system parameters are chosen as follows, $\omega _{c}=1$, $\kappa _{a}=\kappa _{m}=0.1\omega _{c}$, $\gamma _{c}=10^{-5}\omega _{c}$, $G_{a}=G_{m}=0.08\omega _{c}$, $\bar{n}_{c}=0.5$.

### $G_{0}=0$ case

In the beginning of the analysis, we consider the case without effective cavity-magnon interaction,i.e., $G_{0}=0$. This condition can be realized by adopting the appropriate value of $r_{1j}$ and placing the trap at the node of the cavity magnetic field^52,55^. In Fig. 2, we plot the logarithmic negativity of entanglement between two CM motions $E_{n}$ versus the two magnon detuning $\Delta _{m1}$ and $\Delta _{m2}$. It is shown in Ref.^33,56^ that when $\Delta _{a}=\omega _{c}$, the beam-splitter interaction between the cavity field and the CM mode ($\delta a\delta c^{\dagger }_{j}+\delta a^{\dagger }\delta c_{j}$) is promoted. In contrast, as for $\Delta _{a}=-\omega _{c}$, the cavity field and the CM mode can be entangled due to the activated parametric interaction $\delta a\delta c_{j}+\delta a^{\dagger }\delta c^{\dagger }_{j}$. Inspiring by these clues, we analyze the logarithmic negativity of entanglement $E_{n}$ for $\Delta _{a}=\omega _{c}$ in Fig. 2(a) and $\Delta _{a}=-\omega _{c}$ in Fig. 2(b).

**Fig. 3:**
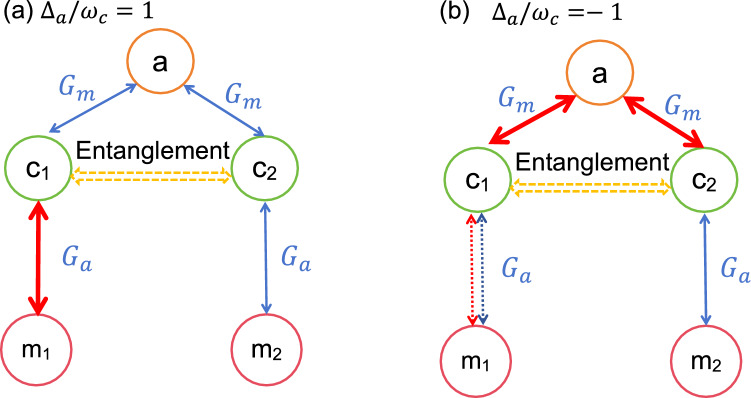
The systematic interaction diagram of the optimal condition of entanglement generation in Fig. 2 for (**a**) $\Delta _{a}/\omega _{c}=1$ and (**b**) $\Delta _{a}/\omega _{c}=-1$. In Fig. 3(a), the magnon detunings are chosen as $\Delta _{m1}/\omega _{c}=-1$, $\Delta _{m2}/\omega _{c}=1$. While in Fig. 3(b) the we have assumed $\Delta _{m2}/\omega _{c}=1$. The blue and red solid lines represent the beam splitter interaction and parametric interaction. The dotted lines in Fig. 3(b) depicts the possible interaction between $c_{1}$ and $m_{1}$.

It can be seen from Fig. 2(a) that for $\Delta _{a}/\omega _{c}=1$ the optimal conditions for entanglement between the two CM modes are almost $\Delta _{m1}/\omega _{c}=-1$, $\Delta _{m2}/\omega _{c}=1$ and $\Delta _{m1}/\omega _{c}=1$, $\Delta _{m2}/\omega _{c}=-1$. However, for $\Delta _{a}/\omega _{c}=-1$, the entanglement can be obtained under the condition $\Delta _{m1}/\omega _{c}=1$ or $\Delta _{m2}/\omega _{c}=1$. To understand the mechanism of the entanglement between the two CM modes more clearly, we plot the interaction diagram for these optimal conditions in Fig. 3. As shown in Fig. 3(a), for $\Delta _{a}=\omega _{c}$, there exist beam-splitter interactions between the cavity field and two CM modes. To realize entanglement in this case, a parametric interaction is required among the system particles. Hence $\Delta _{mj}/\omega _{c}=-1$ is needed for the generation of entanglement in this system. Meanwhile, the entanglement between the two CM modes is very sensitive to the thermal phonons. By inserting the beam splitter interaction ($\Delta _{mj}/\omega _{c}=1, j=1,2$), the thermal phonons can be extracted by the related magnomechanical cooling process. Then the two CM modes can become entangled with the combination of parametric interaction (between $c_{2}$ and $m_{2}$) and beam splitter interaction (between $c_{1}$ and $m_{1}$). Note that we only consider the case $\Delta _{m1}/\omega _{c}=-1$, $\Delta _{m2}/\omega _{c}=1$ in Fig. 3(a). The other case $\Delta _{m1}/\omega _{c}=1$, $\Delta _{m2}/\omega _{c}=-1$ can be analyzed by exchanging the subscript order of two spheres.

In Fig. 3(b), we can see that for $\Delta _{a}/\omega _{c}=-1$ there are parametric interactions between the cavity field and the two CM modes. Then quantum coherence between two CM modes can be established with the help of the intermediate cavity field. To stimulate the obvious entanglement between the two CM modes, we need to reduce the thermal phonons of the CM modes. Thus, the corresponding magnomechanical cooling process (between $c_{j}$ and $m_{j}$), i.e., the beam-splitter interaction for $\Delta _{mj}/\omega _{c}=1$ is demanded.

**Fig. 4:**
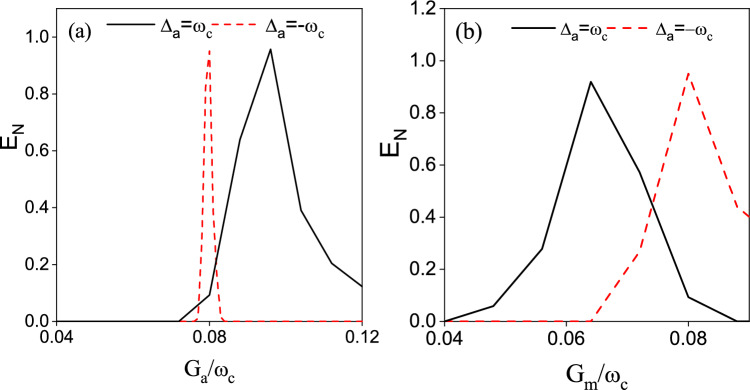
The logarithmic negativity $E_{n}$ versus the normalized coupling coefficient (**a**) $G_{a}/\omega _{c}$ and (**b**) $G_{m}/\omega _{c}$. For Fig. 4(a), we have chosen $G_{m}/\omega _{c}=0.8$, $\Delta _{m1}/\omega _{c}=-0.99$ and $\Delta _{m2}/\omega _{c}=1.03$. While for Fig. 4(b), $G_{a}/\omega _{c}=0.8$, $\Delta _{m1}/\omega _{c}=1.02$ and $\Delta _{m2}/\omega _{c}=-0.38$. Other parameters are the same as in Fig. 2.

To study the effect of the coupling coefficients $G_{a}$ and $G_{m}$ on $E_{n}$, we plot the logarithmic negativity $E_{n}$ versus the normalized frequency $G_{a}/\omega _{c}$ in Fig. 4(a) and versus the normalized frequency $G_{m}/\omega _{c}$ in Fig. 4(b). From Fig. 4(a) we can find that with the increase of the coupling coefficient $G_{a}$ the entanglement between the two CM modes would first increase and then decrease. As seen in Fig. 3(a), the coupling coefficient $G_{a}$ is related to the parametric interaction between $m_{1}$ and $c_{1}$. The coupling coefficient $G_{m}$ is linked to the beam-splitter interaction between *a* and $c_{j}$. To achieve entanglement between $c_{1}$ and $c_{2}$, we need to balance these two effects to a comparable degree. A considerable value of $G_{a}$ would be beneficial for the generation of entanglement between $m_{1}$ and $c_{1}$. However, if the value of $G_{a}$ is much higher than that of $G_{m}$, the restricted value of $G_{m}$ would limit the quantum coherence transfer between *a* and $c_{j}$. Then the establishment of the entanglement between $c_{2}$ and $c_{1}$ would also be restricted. In contrast, for $\Delta _{a}=-\omega _{c}$, the coupling coefficient $G_{m}$ is related to the parametric interaction, and $G_{a}$ is related to the beam splitter interaction. With increasing $G_{a}$, the magnomechanical cooling process is strengthened and the entanglement between $c_{1}$ and $c_{2}$ would be promoted by the reduced thermal phonons. However, without further increasing the parametric interaction (the term related to $G_{m}$) further, the entanglement between $c_{1}$ and $c_{2}$ would be limited.

**Fig. 5:**
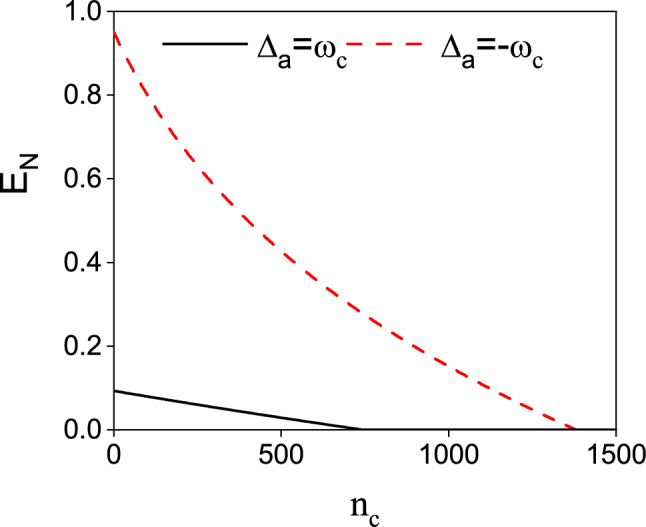
The logarithmic negativity $E_{n}$ as a function of $\bar{n}_{c}$. $G_{a}/\omega _{c}=G_{m}/\omega _{c}=0.8$. Other parameters are the same as in Fig. 4.

The effects of dissipation and decoherence on entanglement and coherence is a significant topic in quantum optics. Following the analysis in Ref.^64–66^, we investigate the effect of thermal occupation $\bar{n}_{c}$ on the logarithmic negativity $E_{n}$ in Fig. 5. As shown in Fig. 5, the entanglement generated in our scheme shows great robustness against the thermal occupation. With increasing thermal occupation $\bar{n}_{c}$, the logarithmic negativity exists even as high as $\bar{n}_{c}=1500$ for $\Delta _{a}=-\omega _{c}$. The thermal range of the existing entanglement is narrower for $\Delta _{a}=\omega _{c}$. This may be due to the fact that for $\Delta _{a}=-\omega _{c}$ and $\Delta _{m1}=\Delta {m2}=\omega _{c}$ there is one more parametric interaction for entanglement generation.

#### $G_{0}\ne 0$ case

**Fig. 6:**
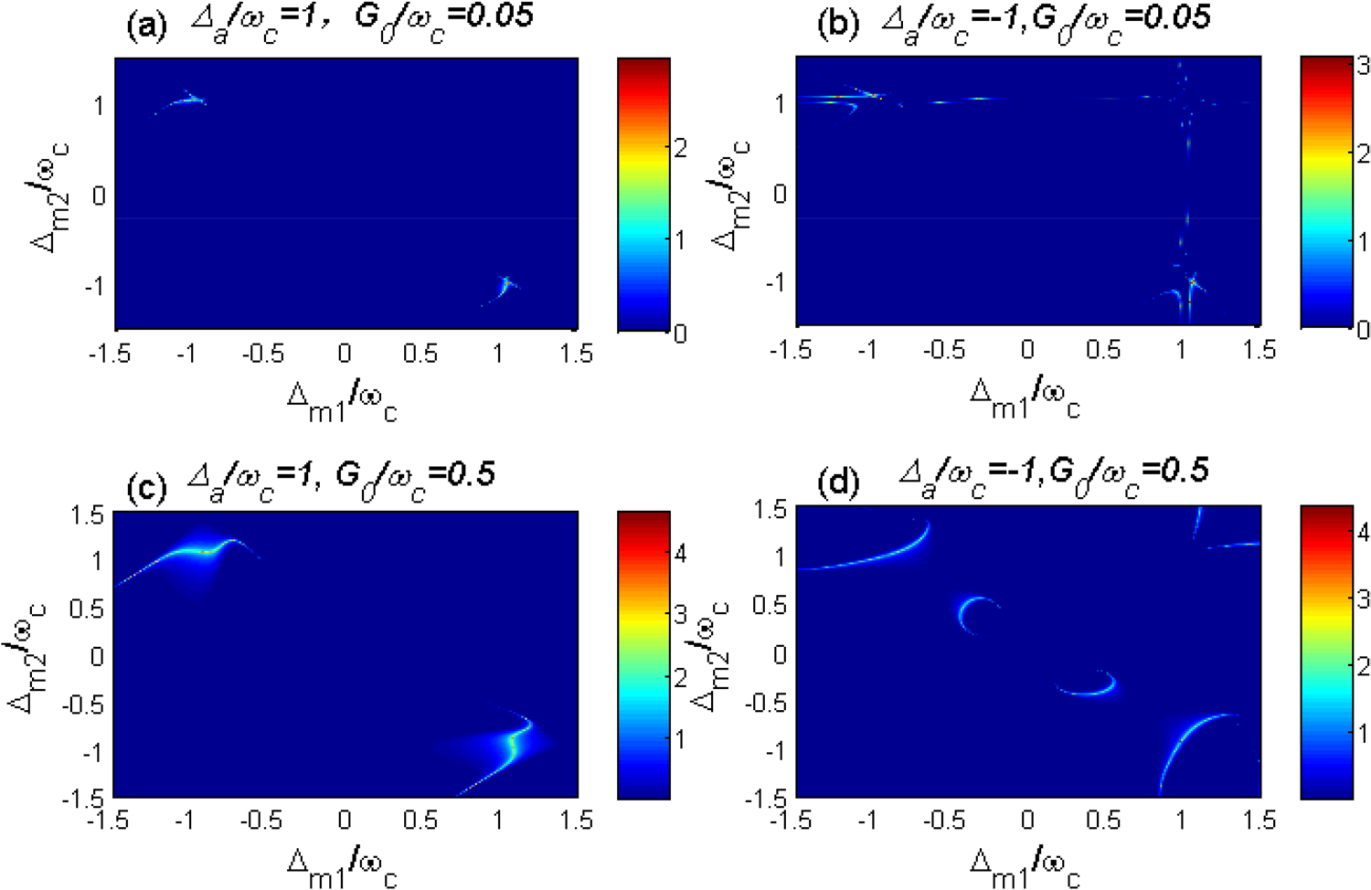
Phase diagram of the entanglement logarithmic negativity between the two CM motions as a function of $\Delta _{m1}/\omega _{c}$ and $\Delta _{m2}/\omega _{c}$ for (**a**) $\Delta _{a}/\omega _{c}=1$ and (**b**) $\Delta _{a}/\omega _{c}=-1$, respectively. Other parameters are the same as in Fig. 4.

**Fig. 7:**
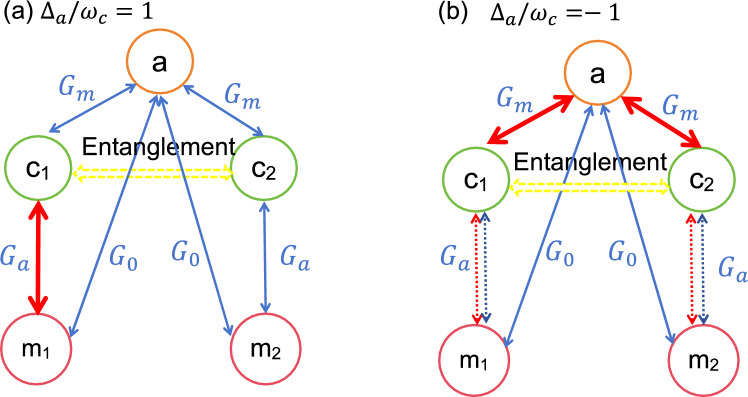
The systematic interaction diagram of the optimal condition in entanglement generation in Fig. 6 for (**a**) $\Delta _{a}/\omega _{c}=1$ and (**b**) $\Delta _{a}/\omega _{c}=-1$. In Fig. 7(a), the magnon detunings are chosen as $\Delta _{m1}/\omega _{c}=-1$, $\Delta _{m2}/\omega _{c}=1$. While in Fig. 7(b) the we have assumed $\Delta _{m2}/\omega _{c}=1$. The blue and red solid lines represent the beam splitter interaction and parametric interaction. The dotted lines in Fig. 7(b) depicts the possible interaction between $c_{1}$ and $m_{1}$.

To investigate the effect of the coupling rate $G_{0}$, we plot $E_{n}$ as a function of $\Delta _{m1}/\omega _{c}$ and $\Delta _{m2}/\omega _{c}$ for different values of $G_{0}$ in Fig. 6. It is shown from Fig. 6(a) and (c) that the areas of nonzero $E_{n}$ for $\Delta _{a}/\omega _{c}=1$ are close to the points $\Delta _{m1}/\omega _{c}=-1$, $\Delta _{m2}/\omega _{c}=1$ or $\Delta _{m1}/\omega _{c}=1$, $\Delta _{m2}/\omega _{c}=-1$. This phenomenon can be explained by the schematic of system interaction in Fig. 7. As shown in Fig. 7(a), for $\Delta _{a}/\omega _{c}=1$, there exists an optomechanical cooling channel through the beam splitter interaction between the cavity field and the CM modes. In addition, the cavity field and the magnon modes are coupled through the beam splitter interaction between them. Under the condition $\Delta _{mj}/\omega _{c}=-1$, there exists a parametric interaction and subsequent quantum coherence between the CM modes and the magnon modes. Then with the help of the intermediate cavity field or magnon modes, quantum entanglement would be established between two CM modes. Hence, the condition $\Delta _{m1}/\omega _{c}=-1$, that is, a parametric interaction between the CM modes and the magnon modes, would be beneficial in establishing entanglement. However, magnon detuning $\Delta _{m2}/\omega _{c}=1$ is related to the magnomechanical cooling channel, which can be used to reduce the thermal occupation of CM modes. For cases $\Delta _{a}/\omega _{c}=1$ and $G_{0}/\omega _{c}=0.05$ (shown in Fig. 6b), entanglement appears almost immediately under the condition $\Delta _{m1}/\omega _{c}=1$ or $\Delta _{m2}/\omega _{c}=1$. However, for $G_{0}/\omega _{c}=0.5$, as seen in Fig. 6(d), the optimal entanglement area has changed into several discontinuous areas. This change is rooted in the interaction between *a* and $m_{1,2}$. When $G_{0}/\omega _{c}=0.05$, the cavity-magnon interaction is relatively small. The entanglement mechanism $\Delta _{a}/\omega _{c}=-1$ is similar to that case of Fig. 2(b). However, for the case $G_{0}/\omega _{c}=0.5$, as shown in Fig. 7(b), the cavity-magnon interaction would affect the magnomehcanical cooling channel. The cavity field and magnon modes act as intermediaries between two CM modes. In other words, thermal phonons could be extracted under different conditions. As a result, the optimal condition for getting nonzero $E_{n}$ is altered.

**Fig. 8:**
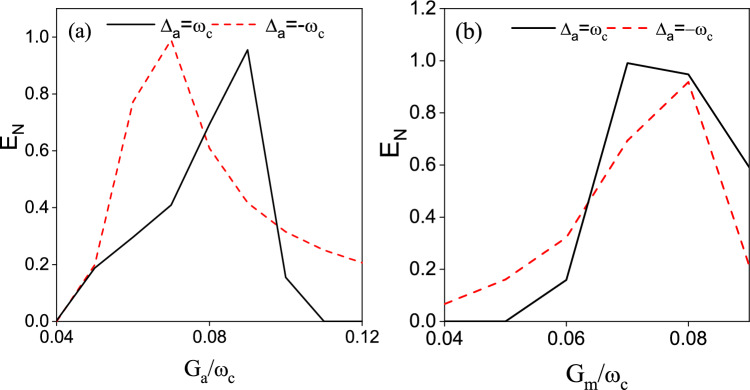
The logarithmic negativity $E_{n}$ for $G_{0}=0.5\omega _{c}$ versus the normalized detuning $\Delta _{m2}/\omega _{c}$ (**a**) with $\Delta _{a}/\omega _{c}=1$, $\Delta _{m1}/\omega _{c}=1$ and (**b**) $\Delta _{a}/\omega _{c}=-1$, $\Delta _{m1}/\omega _{c}=1$. For Fig. 8(a), we have chosen $G_{m}/\omega _{c}=0.8$, $\Delta _{m1}/\omega _{c}=-0.99$ and $\Delta _{m2}/\omega _{c}=1.03$. While for Fig. 8(b), $G_{a}/\omega _{c}=0.8$, $\Delta _{m1}/\omega _{c}=1.02$ and $\Delta _{m2}/\omega _{c}=-0.38$. Other parameters are the same as in Fig. 6.

As shown in Fig. 8, we plot the logarithmic negativity between two CM modes as a function of (a) $G_{a}$ and (b) $G_{m}$. We can see that there appears a similar peak structure as in Fig. 4. Analogous to Fig. 4, these two coupling coefficients are related to the parametric interaction and beam splitter interaction. To gain entanglement between two CM modes, we need to balance these two effects.

**Fig. 9:**
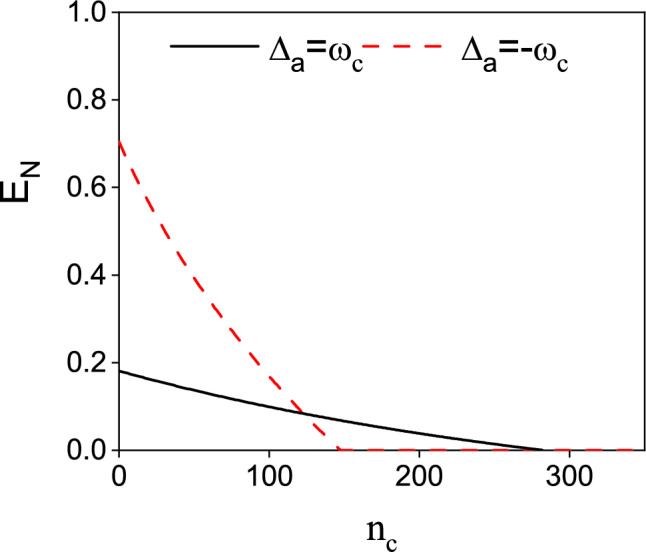
The logarithmic negativity $E_{n}$ for $G_{0}=0.5\omega _{c}$ versus $\bar{n}_{c}$. Other parameters are the same as in Fig. 6.

To study the effect of $\bar{n}_{c}$ on the entanglement between the two CM modes, we plot $E_{n}$ versus $\bar{n}_{c}$ in Fig. 9. As shown in Fig. 9, the existing thermal range for the $\Delta _{a}/\omega _{c}=-1$ case is almost approaching 300. However, for $\Delta _{a}/\omega _{c}=1$, the tolerable thermal occupation is less than 200. We can infer from Ref. ^56^ that with the participation of the cavity-magnon interaction the entanglements between the CM modes and the cavity field (magnon mode) are weakened. Thus, the entanglement between two CM modes is more vulnerable to thermal noise than that in Fig. 5.

## Conclusion

In summary, a scheme for generating entanglement between two macroscopic CM modes is proposed in a double-levitated micromagnet system. The entanglement can be realized by choosing appropriate detunings of the magnon modes. We discuss the generation of entanglement for both cases $G_{0}=0$ and $G_{0}\ne 0$. The effects of system parameters such as the coupling strength and thermal occupation on the entanglement are also analyzed. The research based on our scheme can be applied to further quantum information processing and macroscopic quantum simulation.

## Data Availability

Data supporting the findings of this study are available in this article.
